# Natural selection under conventional and organic cropping systems affect root architecture in spring barley

**DOI:** 10.1038/s41598-022-23298-3

**Published:** 2022-11-22

**Authors:** Md. Nurealam Siddiqui, Michael Schneider, Marissa B. Barbosa, Jens Léon, Agim Ballvora

**Affiliations:** 1 Institute of Crop Sciences and Resources Conservation (INRES)-Plant Breeding, University of Bonn, 53115 Bonn, Germany; 2 Present Address: Department of Biochemistry and Molecular Biology, Bangabandhu Sheikh Mujibur Rahman Agricultural University, Gazipur, 1706 Bangladesh; 3 Institute for Quantitative Genetics and Genomics of Plants, Universitätsstraße 1, 40225 Düsseldorf, Germany; 4 Field Lab Campus Klein-Altendorf, University of Bonn, Klein-Altendorf 2, 53359 Rheinbach, Germany

**Keywords:** Ecology, Genetics, Plant sciences

## Abstract

A beneficial root system is crucial for efficient nutrient uptake and stress tolerance. Therefore, evaluating the root system variation for breeding crop plants towards stress adaptation is critically important. Here, we phenotyped root architectural traits of naturally adapted populations from organic and conventional cropping systems under hydroponic and field trails. Long-term natural selection under these two cropping systems resulted in a microevolution of root morphological and anatomical traits. Barley lines developed under an organic system possessed longer roots with narrow root angle, larger surface area, increased root mass density, and a thinner root diameter with an increased number of metaxylem vessels. In contrast, lines adapted to the conventional system tend to have a shorter and wider root system with a larger root volume with a thicker diameter but fewer metaxylem vessels. Allometry analysis established a relationship between root traits and plant size among barley genotypes, which specifies that root angle could be a good candidate among studied root traits to determine root-borne shoot architecture. Further, multivariate analyses showed a strong tendency towards increased variability of the organically adapted population's root morphological and anatomical traits. The genotyping of ancestor populations validated the observations made in these experiments. Collectively, this results indicate significant differences in root phenotypes between conventional and organic populations, which could be useful in comparative genomics and breeding.

## Introduction

Historically, crop improvement programs heavily focused on the role of above-ground plant functional traits for potential adaptation in specific abiotic stresses^1–3^. While much research was performed on the above-ground parts of the plant, the below-ground part is often neglected, although their improvement is indirect in plant breeding programs^4,5^. Recently, the challenges in increasing food production with minimal environmental impact led to a renewed interest in understanding and improving the plant root system in terms of root architectural and anatomical attributes. Both root architectural and anatomical features are critical for plant endurance under climatic instability and nutrient insufficient conditions^6–8^.

The crucial role of roots for productivity and adaptability depends on the root system architecture– the collective term for the spatial and temporal arrangement of all root parts and root structural features arising from a single plant^9^. Root system architecture is dynamic and exhibits a high degree of plasticity in response to changing growing conditions such as soil moisture, soil structure and composition, availability of nutrients, and the below-ground competition^4,9^. The differences in root system architecture or root morphological characteristics were reportedly known to influence plants' competitive ability for soil resources, enabling them to respond and thrive in different agricultural systems^9–12^.

Barley (*Hordeum vulgare* L.) is a self-pollinating crop species under the Gramineae family and tribe *Triticeae* where two other evolutionary related grains crops wheat and rye, belonged^13,14^. Its importance has been recognized since the start of civilization and was considered one of the earliest grain crops in the world^15^. Now, barley is flourishing as a significant crop globally and ranks fourth after maize, wheat, and rice in terms of its area of cultivation and total production^16^. It is grown across several geographical regions worldwide, and because of its versatility and adaptability, this crop is successfully grown even in adverse agro-ecological conditions^17^. The crop is being utilized for it several essential uses, including animal feed and fodder, malt production used in brewery and distillery industries, and biofuels which is important as a source of renewable energy^18–21^, and still remain a staple food crop in some areas in the world, particularly in developing countries^22^.

In agricultural research, long-term selection experiments served as an important platform that could provide essential information on the performances and adaptability of plants and the sustainability of the cropping systems^23,24^. While several studies investigated the impact of long-term selection experiments in crops such as maize23, wheat25, and legumes24, these studies focused on the above-ground traits and highlighted evaluating yield and yield stability, yield trends and sustainability, and biomass productivity and nutrient cycling^24^. However, the effects of natural adaptation to different cropping systems, particularly allele frequency changes, have only been investigated in above-ground architecture. The effect of long-term adaptation on root system architecture and the anatomy of economically important crops remains largely unknown. In the perspective of the potential of low-input agriculture farming for sustainable crop production, further investigation is essential.

The root system architecture of barley populations modifies over time due to selection pressure by farming systems varying in the application of different types and amounts of chemical fertilizer. Therefore, we hypothesize that barley populations, adapting twenty years towards organic cropping system (OCS) and conventional cropping system (CCS) establish variant root systems, promoted by the natural selection within the populations. To address this hypothesis, root phenotyping was performed on barley lines that were grown for twenty consecutive years in either an organically or conventionally managed cropping system. In this study, the major objective was therefore to combine and compare root anatomy and architecture responses and explore the effect of OCS and CCS on genetic variation in root traits. In detail, we formulated the following objectives: (i) root-shoot phenotypic characterization of the CCS and OCS populations by investigating randomly selected subsets of these two naturally adapted lines, (ii) measure the root architectural traits in a hydroponic environment where the entire root system extracted at the seedling stage, (iii) phenotype the root system traits attributes under field conditions, and (iv) compare the root morphology between CCS and OCS adapted lines to estimate the overall variation of root phenotypes.

## Materials and methods

### Plant materials

Root phenotyping was performed in two barley BC_2_F_23_ populations, originating from an identical founder population was developed by the Department of Plant Breeding, University of Bonn, Germany. The crossing scheme and the cultivation practices were described by^26^ and provided in Fig. S1. The population was derived from the initial cross of the cultivar Golf (*H. vulgare* L. *ssp. vulgare*) and the wild form ISR42-8 (*Hordeum vulgare* L. *ssp. spontaneum*) Golf was selected as an elite cultivar and used as the maternal plant in the initial barley cross, while the wild-form was used as the donor to increase the genetic diversity of the population (Fig. S1A). A single F_1_ plant was than back-crossed to the cultivar to retain only a single wild-form allele per locus, and six BC_1_F_1_ plants were randomly selected and back-crossed for a second time with Golf. Two plants of each BC_2_F_1_, in a total of 12 sublines, were used to produce 296 BC_2_F_2_ progenies. All crossings were performed in a greenhouse under controlled conditions to avoid admixtures. An equal number of obtained seeds (BC_2_F_3_) from each BC_1_ derived (sub)cross in BC_2_F_2_ were then sown to ensure that all the BC_2_F_2_ introgression lines had equal contributions of wild-form alleles for the following evolutionary adaptation process. According to^27^, the selected plant allocation (plant number in F_1_, BC_1_F_1_, BC_2_F_1_ etc.) in back-crossing leads to a proportion of less than 5% fixation of recurrent alleles in the initial BC_2_F_3_ population.

The produced seeds of the BC_2_F_3_ generation were used to establish the identical population under conventional and organic farming practice. Across the period of 20 generations, harvested seeds were used to establish next year’s population. In this process, the organic and conventional seed material was stored separately from another. We used the seeds from the organic environment to establish the next generation in the OCS environment and analogously proceeded in the CCS environment. No intended artificial selection was applied to the OCS and CCS populations across the entire 20 generations. Therefore, the cropping systems lead to changes in the allele frequency, and with that also alters the phenotypic constitution of the populations. The natural selection process occurred under OCS and CCS for 20 generations (1999–2019) at faculty's experimental station of Campus Klein-Altendorf, University of Bonn. The cultivation practices differed in the two farming systems as under organic conditions; a wide crop rotation was employed with no application of agrochemicals such as herbicides, pesticides and mineral fertilizers, while vice-versa under conventional conditions (Tables S1A and S1B). The average and standard deviation soil nutrient profiles from 2014 to 2020 are provided in Table S2. In total, three hundred (300) and two hundred (200) lines were randomly selected for hydroponic and field root phenotyping, respectively. The experiments conducted both hydroponic and field conditions in accordance with relevant guidelines and regulations of Germany.

### Hydroponic experiment

Three hundred barley lines, 150 evolved under long-term selection in CCS and OCS each, were tested in a hydroponics experiment. The parental lines of these barley populations, Golf and ISR 42-8 were randomly planted six times in each group. Prior to sowing, barley seeds were put in the oven for drying at 40 °C for 24 h to improve the emergence rate. Two seeds per line were sown in each PVC tube (4.5 cm diameter × 45 cm depth) and both parental lines were randomly sown with three replications in each container. The seeds were germinated *in situ* in the PVC tubes containing Aquagran Filterquartz, 2–3.15 mm (Euroquarz GmbH, Dorsten, Germany) with tap water. In the case of both seeds germinating, one was selected for the experiment and the second plant was removed from the tube. The two groups were grown in separate hydroponic containers (76 cm × 59 cm x 41 cm), which were placed 80 cm above floor level next to each other. An additional container of the same size was used as a water and nutrient reservoir. A hydroponic water pump was used and immersed into these containers to periodically flood the plants with water and nutrients from the reservoir into the growing container through the hose connected between them. During planting, the reservoir boxes were filled with tap water, and a day after, the containers were filled with a balanced nutrient solution containing macro and micronutrients^28^. Then pH was adjusted to 5.9–6.0 using diluted NaOH and HCl and adjusted every second day. The water and nutrient solution were renewed once in a week to prevent nutrient exhaustion. Plants were grown in a greenhouse at the Campus Poppelsdorf, University of Bonn, Germany with an 18/12 °C temperature regime for 6 weeks at a photoperiod of 16 h supplemented with artificial lighting to maintain a minimum light intensity of 250 μmol quanta m^−2^ s^−1^. The seedling emergence date was estimated in each genotype at 7 days after seed sowing.

Forty-five days after planting, plants were harvested for the shoot and root measurements. Each tube was taken singly from the growing box and shaken gently to remove the grow substrate without destroying the roots. The whole plant was carefully pulled out from the tube and shoot measurements were manually performed. These include the number of tillers and number of leaves, shoot height (which was measured from the root-shoot junction to the tip of the longest fully expanded leaf), and root length—measured from the hypocotyl to the root tip were gathered using a metric ruler. At approximately 0.5 cm from the root-shoot junction, the shoots were cut to separate the roots for further measurements.

### Field experiment

Two hundred barley lines, 100 developed from long-term selection in CCS and OCS each, were used in the field experiment. The field evaluation was conducted from March to June 2020 at Campus Klein-Altendorf research facility (50° 370′ N, 6° 590′ E), University of Bonn, Germany. The experiment was conducted under rainfed conditions on a homogenous Luvisol with a high field capacity (approximately 25%) and cation exchange capacity (CEC) and soil nutrient profiles and precipitation data are available in Table S2. The genotypes (individual lines within the population) from each of the two different populations were used for the field experiment. Both parental genotypes, Golf (cultivated variety) and ISR 42-8 (wild form) were included as respective control of each population derived under CCS and OCS. The experiment was carried out in a trial measuring 18 m × 12.6 m. This was divided into several plots, where each plot size was 1.5 m × 1.12 m. Each of these was subdivided into six rows, the barley cultivar Scarlett was sown in the two border rows, while ten seeds of each line were sown in each inner four rows. The plant's distance within a row was 14.5 cm, while the distance between rows was 21 cm. The two population groups were grown in different sub-nurseries, and 2 columns of plots planted with Scarlett were used as the borders to separate the two groups and the borders of the experimental area. No additional fertilizer was applied to the trial, with on-demand pest control applied whenever necessary.

Roots were analyzed using "Shovelomics" approach^12,29^, followed by manual phenotyping. Three representative plants of more or less similar in shoot architecture in each line were selected and harvested at the complete flowering stage BBCH51 (Biologische Bundesanstalt, Bundessortenamt und Chemische Industrie^30^. In order to remove the soil debris, roots were rinsed with clean water to further remove the remaining soil particles. Root angle (^0^) was determined as the angle between the two outermost seminal roots from the main shoot were then measured by a phenotyping board with a large protractor^12^. Afterward, root samples were preserved in 70% alcohol (v/v) for image acquisition and morphological trait quantification.

### Root image acquisition and processing using WinRHIZO

To quantify root morphological traits, root samples stored in 70% alcohol were placed in a plexiglass scanner tray (20 cm × 30 cm) with a 3–4 mm deep layer of water. They were adjusted to help untangle the roots and minimize overlapping and aligned vertically on the scanning plates. Thereafter, roots were scanned with a high-resolution Epson scanner (Perfection LA24000) at 600 dots per inch, giving an eight-bit grayscale image. The entire root system rather than a subsample of the root was scanned to avoid errors associated during root sampling. The captured images were subsequently used for root analysis which was performed using WinRHIZO Regular (version 2020a, Regent Instruments Inc., Quebec, Canada). The WinRHIZO software's output gave the following measurements: root length (RL), root volume (RV), root average diameter (RAD), root surface area (RSA), and the number of root tips and forks. The variables calculated as described by^31^ were as follows: root tissue mass density (RMD) as the ratio of RV and root dry weight (RDW). After subsequent analysis, root samples were then oven-dried at 70 °C for 48 h to record the RDW. Trait descriptions with acronyms and units are provided in Table 1.

**Table 1:** List of studied and derived phenotypic traits with trait acronym and unit.

Traits	Trait acronym	Unit
**(A) Shoot morphological traits**		
Date of emergence	DE	Days after sowing (DAS)
Plant Height	PH	cm
Tiller Number	TN	plant^−1^
Leaf Number	LN	plant^−1^
Shoot dry weight	SDW	g plant^−1^
**(B) Root morphological traits**		
Root length	RL	cm
Root surface Area	RSA	cm^2^
Root volume	RV	cm^3^ plant^−1^
Root average diameter	RAD	mm
Root length to volume	L/V	–
Specific root length	SRL	cm/g
Root mass density	RMD	g/cm^3^
Number of tips	#tips	plant^−1^
Number of forks	#forks	plant^−1^
Root angle	RA	^0^
Root dry weight	RDW	g plant^−1^
Root to shoot ratio	R:S	–
**(C) Root anatomical traits**		
Root cross-section area	RXA	mm^2^
Late metaxylem number	LMXN	plant^−1^
Average Late metaxylem area	ALMXA	mm
Total cortical area	TCA	mm^2^
Stele area	SA	mm^2^
Aerenchyma area	AA	mm^2^

### Root anatomical study

To analyze root anatomical parameters, nodal root parts of the main shoot axis at approximately 5–7 cm from the root-shoot junction were selected. The selected root samples were then fixed in 70% (v/v) alcohol until further analysis. Cross-section slides were made by cutting the fixed samples by hand with a sharp razor blade (Apollo, HERKENRATH Solingen, Germany). Thereafter, cuttings were placed on a glass slide, and root cross-section images were taken using a digital microscope (Keyence's VHX-1000D, Germany) with 100X magnification. At least three to five root images per genotype were considered for measuring root anatomical traits. Acquired images were then analyzed by *ImageJ* software^32^, which measured anatomical parameters such as root cross-section area (RXA), total cortical area (TCA), stele area (SA), aerenchyma area (AA), late metaxylem number (LMXN) and average late metaxylem area (ALMXA). Trait descriptions with acronyms and unit are provided in Table 1.

### Statistical analysis

Statistical analyses were performed using R version 4.0.1 (R core development team). For data obtained under hydroponics growing conditions, one-way ANOVA was used to evaluate phenotypic differences between the OCS and CCS populations and their parental lines. Means in each group were compared based on Tukey's HSD using R package agricolae version 1.3–3 at 95% family-wise confidence interval. Pearson correlation analysis was performed on corresponding morphological traits between the two groups under the two growing conditions using 'corrplot' package in R studio. Additionally, an allometric test for similar tissue development was performed by the 'npregfast' R package^33^. A principal component analysis (PCA) using 'PCAtools'^34^ in R package version 2.0.0 was performed to access the phenological diversification between both CCS and OCS. Box- and density plots were generated with the package 'ggpubr' (version 0.4.0), where the compare means extension was implemented as a simple t-test to compare the groups.

### Comparison of root phenotypes with the genomic background

We included the genomic data of the examined populations, presented by^26^ to validate the observed phenotypic variations between the populations. From the entire data set, we extracted the median allele frequency of 18 selected QTL loci, previously described by^35^ to have a functional effect on the root length, the root dry weight, and the root volume. First, we clustered the observed allele frequencies across all generations (3rd, 12th, 16th, 22nd, 23rd) of the same trait (Root length, dry weight, volume) and compared the OCS and CCS by a Bonferroni adjusted T-test. Furthermore, we investigated the allele frequency patterns of these three groups and all 18 QTL loci separately across the generations to observe targeted selection. Finally, the patterns were matched with the phenotypic data, and similarities and variations were described.

## Results

### Root morphological traits

**Figure 1:**
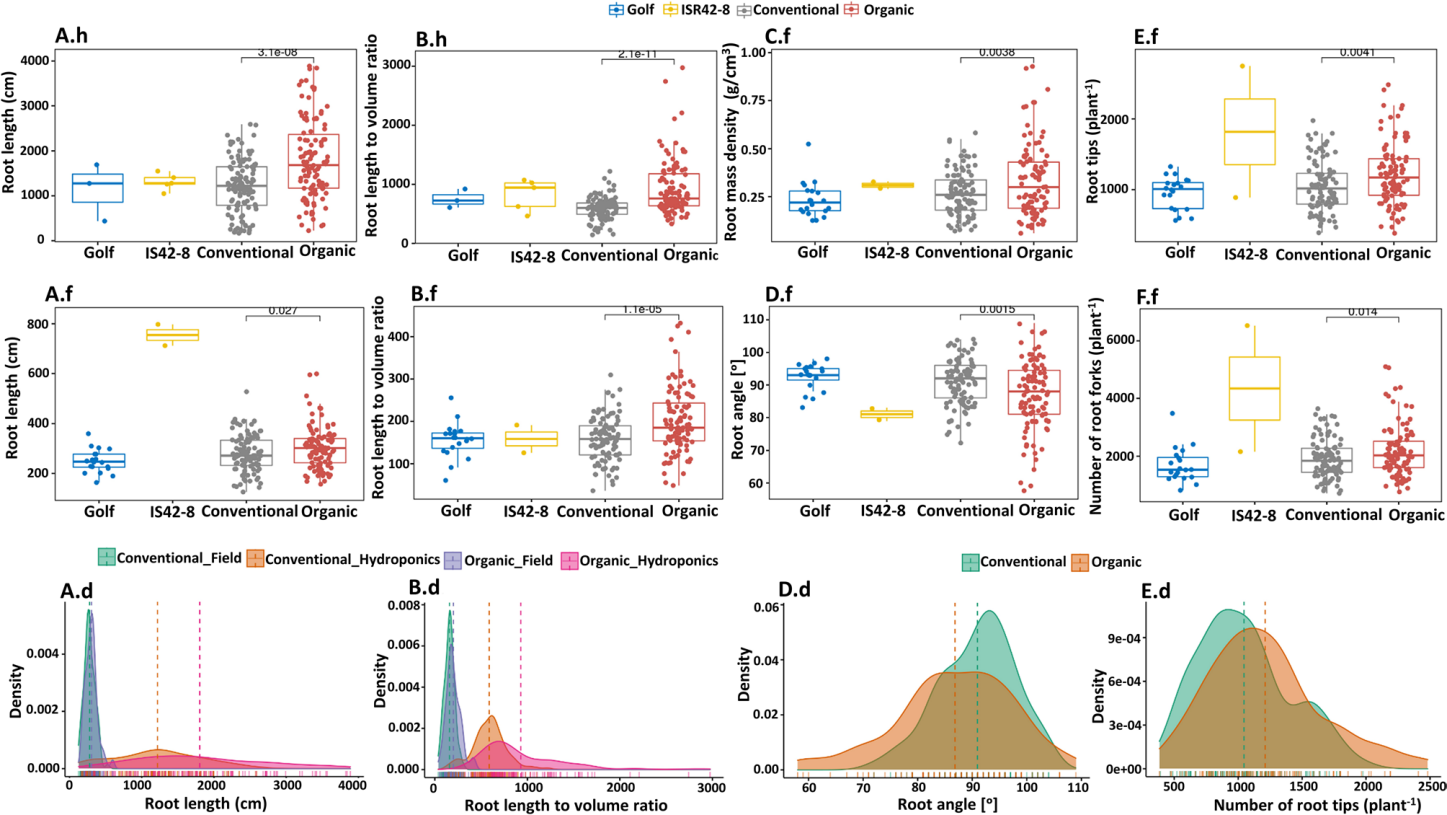
Significantly variant root morphological phenotypes. Boxplots illustrate the overall distribution of observed data points for the parents Golf and ISR 42-8 as well as for the conventional (CCS) and organic (OCS) lines. Density plots highlight the overall distribution of organic and conventional adapted lines. (**A**)—Root length (RL)—the sum of all roots harvested in millimeters (mm), illustrated for all four groups. (**A.h**)—root length measured in the hydroponic experiment; (**A.f**)—field experiment; A.d—distribution histogram for root length in both field and hydroponic experiment for CCS and OCS adapted lines. (**B**)**—**the ratio of root length to volume (L/V). Data available for hydroponics (**B.h**), field (**B.f**), and distribution of the ratio of root length to volume illustrated in B.d. (**C.f**)—Root mass density (RMD) from the field; (**D.f**)—Root angle (RA) from the field, distribution of the root angle illustrated in (**D.d**); (**E.f**)—root tip per plant count from the field, corresponding histogram visualized in (**E.d**). (**F.f**)—root fork per plant count from the field.

The wild-type parent ISR42-8 produced longer root length (RL) than the modern cultivar parent Golf and tested lines (Table S2, Fig. 1A.h,A.f [h = hydroponic; f = field]). The tested lines of the two evolving barley populations displayed significant variations under hydroponic conditions. Barley lines evolved under OCS had on average 3484 mm longer roots compared to CCS under hydroponic treatment (Fig. 1A.h, Table 2). Complementary results under field conditions show as well higher RL for the OCS lines, even though the variance was significantly less pronounced (Fig. 1A.f). In addition, a less evident variance was observed in the field within both groups compared to the hydroponic (Fig. 1A.d). Across both experimental setups, the observed range of RL was higher in the OCS lines [Standard deviation (SD)_OCS_ = 883, SD_CCS_ = 597] (Table 2).

**Table 2:** Comparison of organic and conventional population root phenotypes under field and hydroponic evaluation.

Field									Hydroponics							
Traits	Confidence interval				Average		Standard deviation		Confidence interval				Average		Standard deviation	
	Difference*	Lower	Upper	Adj P value	Conventional	Organic	Conventional	Organic	Difference*	Lower	Upper	Adj P value	Conventional	Organic	Conventional	Organic
RL	25.260	−3.177	53.697	0.101	277.949	303.209	74.652	84.043	584.480	326.313	842.647	0.000	1218.619	1803.099	597.024	883.183
L:V	42.621	18.875	66.367	0.000	158.340	200.961	52.986	77.172	341.173	220.528	461.818	0.000	587.763	928.936	187.054	455.495
RMD	0.065	0.009	0.121	0.015	0.270	0.335	0.117	0.188	−0.003	−0.035	0.029	0.996	0.199	0.196	0.106	0.078
RA	−4.100	−7.279	−0.922	0.005	90.949	86.848	6.915	10.600	**–**	**–**	**–**	**–**	**–**	**–**	**–**	**–**
RSA	−0.526	−8.546	7.494	0.998	79.231	78.705	18.910	24.077	24.433	−10.254	59.120	0.265	186.857	211.290	83.372	114.633
RAD	−0.091	−0.163	−0.018	0.007	0.964	0.873	0.197	0.203	−0.008	−0.046	0.029	0.938	0.407	0.399	0.100	0.118
#Tips	165.890	19.043	312.736	0.020	1046.077	1211.967	357.145	440.106	**–**	**–**	**–**	**–**	**–**	**–**	**–**	**–**
#Forks	280.142	−15.44	575.725	0.070	1903.161	2183.303	680.414	884.413	**–**	**–**	**–**	**–**	**–**	**–**	**–**	**–**
SRL	59.392	−41.66	160.448	0.426	642.498	701.890	234.757	316.895	1577.189	−582.9	3737.3	0.235	3922.056	5499.245	2554.242	8597.680
RV	−0.228	−0.480	0.024	0.091	1.923	1.695	0.681	0.700	0.216	−0.235	0.666	0.603	2.121	2.337	1.053	1.530
RDW	0.041	−0.065	0.147	0.747	0.504	0.545	0.252	0.335	0.025	−0.095	0.146	0.947	0.452	0.478	0.359	0.342
R:S	**–**	**–**	**–**	**–**	**–**	**–**	**–**	**–**	−0.080	−0.151	−0.010	0.019	0.388	0.308	0.246	0.154
SA	−0.009	−0.027	0.009	0.537	0.230	0.221	0.042	0.057	0.002	−0.013	0.017	0.991	0.104	0.106	0.044	0.043
AA	−0.054	−0.093	−0.016	0.002	0.341	0.287	0.110	0.099	−0.005	−0.006	−0.004	0.000	0.041	0.036	0.004	0.003
ALMXA	0.000	0.000	0.000	0.288	0.002	0.002	0.000	0.001	−0.001	−0.001	0.000	0.001	0.003	0.003	0.001	0.001
LMN	0.417	0.128	0.705	0.001	4.755	5.172	0.733	0.858	−0.258	−0.575	0.059	0.154	5.540	5.282	0.936	0.900
RXA	−0.129	−0.213	−0.045	0.001	1.053	0.924	0.237	0.227	0.005	−0.035	0.045	0.988	0.308	0.313	0.111	0.121
TCA	−0.120	−0.192	−0.048	0.000	0.891	0.771	0.210	0.186	0.004	−0.025	0.032	0.988	0.204	0.207	0.079	0.087
DE	**–**	**–**	**–**	**–**	**–**	**–**	**–**	**–**	0.951	0.511	1.392	0.000	6.221	7.173	1.259	1.305
PH	**–**	**–**	**–**	**–**	**–**	**–**	**–**	**–**	3.118	0.552	5.684	0.010	56.991	60.109	7.187	7.570
LN	**–**	**–**	**–**	**–**	**–**	**–**	**–**	**–**	2.473	−0.846	5.792	0.219	17.894	20.367	8.252	10.880
TN	**–**	**–**	**–**	**–**	**–**	**–**	**–**	**–**	−0.298	**–**1.195	0.600	0.827	5.088	4.791	2.651	2.588
SDW	**–**	**–**	**–**	**–**	**–**	**–**	**–**	**–**	0.312	0.011	0.612	0.039	1.208	1.520	0.685	1.035

Tukey HSD pairwise comparison for root morphology, anatomy and shoot-related traits at 95% confidence level showing the difference between group means (second column) and the adjusted *p*-value (fifth column) for all root traits (first column) between the two groups of barley offspring lines. Besides, the average value and standard deviation of the values for ISR42-8 and Golf are illustrated. The stable is split in two main windows—one for field, the other for hydroponic experiment. *Organic always as first value—negative values are related to smaller in organic lines. See Table 1 for trait description.

The root length to volume (L/V) is an important indicator of the soil volume that can be explored by the roots. Under hydroponics conditions, variations were found for L/V between the parental genotypes as well as between the OCS and CCS populations (Tables 2 & S3). The organic lines were characterized by a significantly higher L/V, indicating a much more distinct exploration of the soil by these lines (Fig. 1B.h,B.f). In comparison to field, highest diversity in L/V was found under hydroponic experiments within both OCS and CCS populations (Fig. 1B.d).

The root mass density (RMD) is the ratio of root volume for a given root mass and is a key indicator of root thickness. Although significant variations existed between ISR42-8 and Golf under hydroponics conditions, such significant variations were not found between the OCS and CCS groups (*P* = 0.09) (Fig. 1C.f and Tables 2 and S3).

The root angle (RA) measurements were only performed under field conditions since plants grown under hydroponics conditions were placed in uniform growing vessels and the direction of root growth is restricted by tubes. Significant variation was observed for the RA between the two parental lines, which was also reflected in the CCS and OCS lines (Fig. 1D.f). ISR42-8 was characterized by an 11.5° average narrower RA than Golf (Table S3). The RA was 4.1° bigger in the OCS compared to the CCS population (*P* = 0.005) (Table 2). However, a higher diversity in RA was observed in the OCS compared to the CCS lines (Fig. 1D.d, Table 2).

In addition to the RA, the number of root tips and forks was measured under field conditions only. Both tips and forks indicate a similar pattern, where the OCS lines produced on average more for both *P*_Forks_ = 0.014, *P*_Tips_ = 0.0041 (Fig. 1E.f,F.f). After applying a *P*-adjustment, the number of forks count was no longer significantly different between OCS and CCS (*P*_Forks_ = 0.07, Table 2*)*. Complementary, ISR 42-8 was observed to produce more tips and forks than Golf, which remained highly significant even after probability adjustment (Fig. 1E.f,F.f, Table S3). The distribution and the standard deviation of observed phenotypes highlight once more the fact that the OCS lines tend to have a higher variation (Fig. 1E.d, Table 2). Similarly, a significant increasing trend was recorded in root surface area (RSA) and root average diameter (RAD) by ISR42-8 as compared to Golf under hydroponics (Table S3). Contrasting to the parental genotypes, no variation was observed between OCS and CCS lines for RSA (Table 2).

### Root anatomical traits

**Figure 2:**
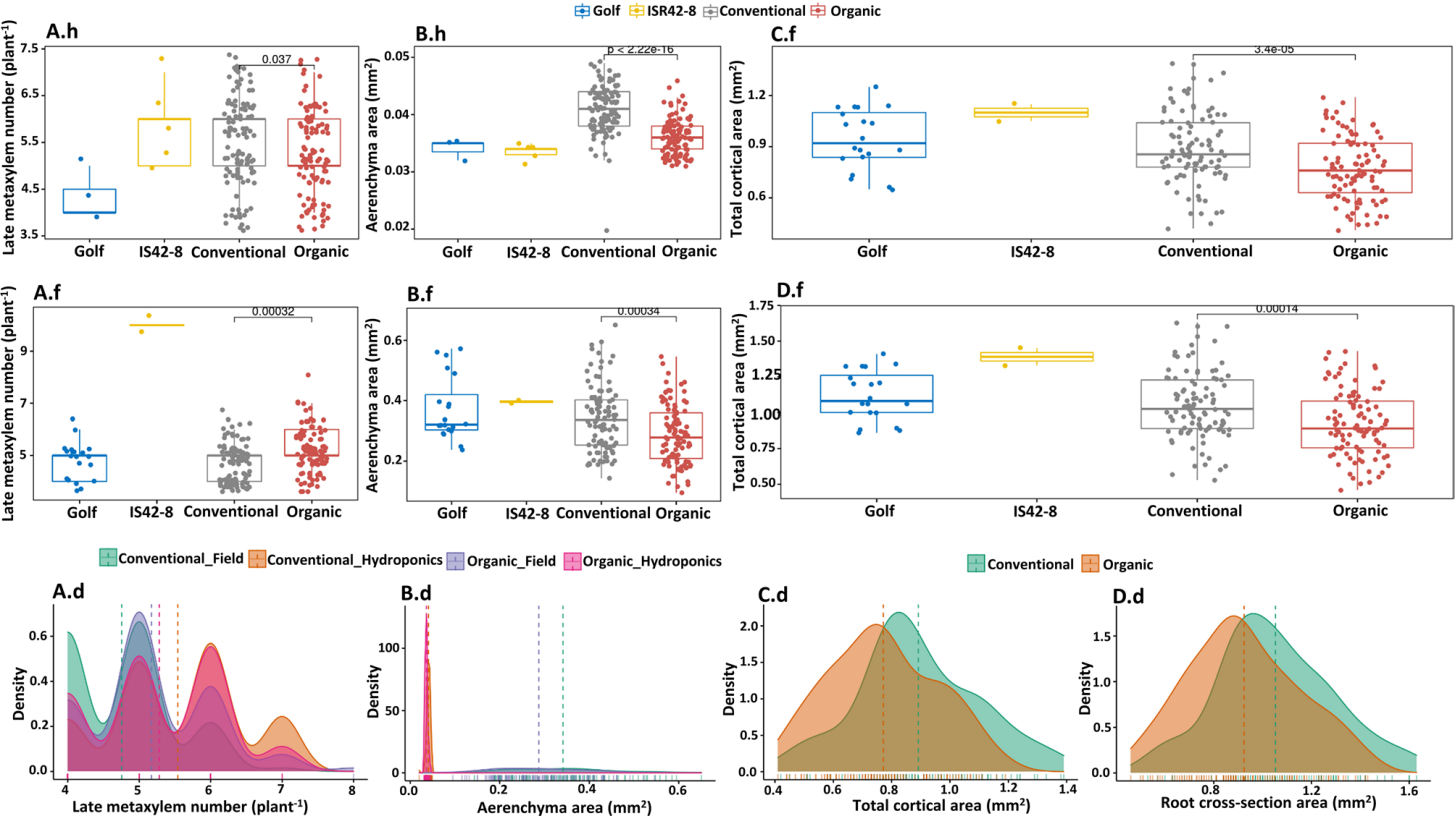
Significantly variant root anatomical traits. Boxplots illustrate the overall distribution of observed data points for the parents Golf and ISR 42-8 as well as for the conventional (CCS) and organic (OCS) lines. (**A**) –Late metaxylem number (LMN)—the sum of all roots harvested and expressed by plant^−1^, illustrated for all four groups. A.h—Late metaxylem number measured in the hydroponic experiment; A.f—field experiment; A.d—distribution histogram for late metaxylem number in both field and hydroponic experiment for CCS and OCS adapted lines. (**B**)**—**Aerenchyma area (AA). Data available for hydroponics (B.h), field (B.f), and distribution of the aerenchyma area illustrated in (**B.d**). (**C.f**)—Total cortical area (TCA) from the field; (**D.f**)—Root cross-section area (RA) from the field, distribution of the total cortical area and root cross-section area illustrated in C.d and D.d, respectively.

Within the observed anatomical traits, four were considered due to their relevance and variation between the systems. In both hydroponic and field experiments, significant variations were observed for the late metaxylem number (LMXN) between the parental lines as well as OCS and CCS lines (Tables 2 & S3, Fig. 2A.h,A.f). An increased LMXN for ISR 42-8 compared to Golf was observed (Fig. 2A.h). Regarding the CCS and OCS lines, a heterogenic scenario was presented over both experimental setups. While the median LMXN under CCS was identical with ISR 42-8 in the seedling stages of plant development (Fig. 2A.h), it was much lower in flowering stages under field conditions (Fig. 1A.f). Additionally, the LMXN was significantly higher in the CCS lines in the seedling stage compared to OCS lines, vice-versa LMXN was observed at the flowering time point (Fig. 2A.d).

The intercellular space, represented by the aerenchyma area (AA), was observed to be significantly more pronounced in the tested CCS compared to OCS lines in both environments (Fig. 2B.h,B.f). Furthermore, the OCS population did not show significant differences to both parents under hydroponics conditions, however, when grown under field conditions, it was noted that Golf had a significantly higher AA mean value as compared to the OCS population (Table S3). As illustrated by the values, the AA expended from early to late stages by a magnitude of 10-folds (Fig. 2B.d). In general, although the two parents did not indicate phenotypic variations, OCS and CCS lines showed significant variations (Table S4).

A 0.12 mm^2^ decreased average total cortical area (TCA) was recorded in the OCS compared to the CCS population under field conditions (*P* = 0.003, Fig. 2C.f), although substantial variations for TCA was observed within OCS and CCS populations (Fig. 2C.d). The root cross-section area (RXA) is a two-dimensional axis of the root which is an important indicator of root thickness. In the hydroponic examination of the seedling stage, significant variations existed between the CCS and ISR42-8 as well as OCS population (Tables 2 and S3). The complementary study under field conditions observed a noticeable variation for OCS from both parental genotypes and the CCS (Table S3). About 0.13 mm^2^ increased average value for RXA was identified for CCS (Fig. 2D.f), while consistent significant variations were also observed between the populations in the under field experiment, where 0.13 mm^2^ increased average value for RXA was identified for CCS (Fig. 2D.f). Analog (Fig. 2D.d). Analogue to the AA, the RXA indicates a lower root extension in the OCS compared to the CCS population. For the stele area (SA), significant variations were only observed at the flowering stage, where ISR42-8 generally had the highest SA and varies significantly between Golf and its progeny lines (Tables 2 and S3).

### Shoot-related traits

**Figure 3:**
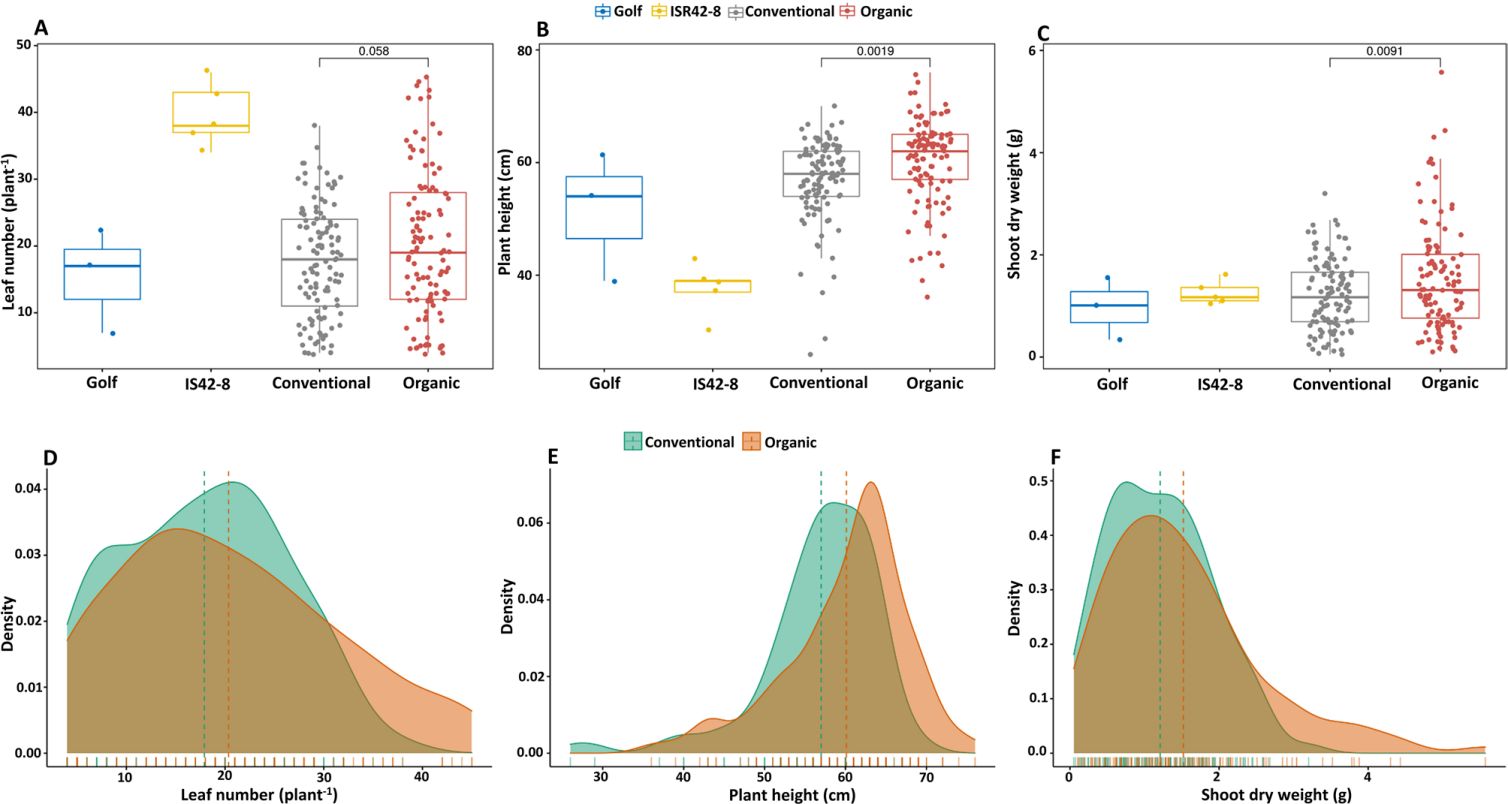
Above-ground plant characteristics. Boxplots illustrate the overall distribution of observed data points for the parents Golf and ISR 42-8 as well as for the conventional (CCS) and organic (OCS) lines under the hydroponic experiment. (**A**)—Leaf number (LN) expressed by; (**B**)—Plant height (PH) and C-Shoot dry weight (SDW). The data distribution of the leaf number, plant height and shoot dry weight is illustrated in (**D**,**E**,**F**), respectively.

Beyond the root-related phenotypic observations, above-ground characteristics were also recorded to assess the root-borne shoot dynamics (Figs. 3 and S2). Among the OCS and CCS populations and the parents, ISR42-8 had the longest duration of emergence. While CCS-adapted lines took on average 5.8 days of emergence (DE), OCS-adapted lines emerged 1.8 days later (7.6 days) (Fig. S2). No variation was observed for the tiller number (TN) throughout all tested groups, while ISR 42-8 tends to produce much more leaf number (LN), accompanied by a lower plant height (PH) and higher shoot dry weight (SDW) (Table S4, Fig. S2). The OCS and CCS plants significantly differed in PH as well as SDW (Fig. 3B,C). The LN was marginally above the probability threshold of 0.05 (*p* = 0.058, Fig. 3A), with a clear tendency of increased variability in phenotypic variation (Fig. 3D). Similar trend was recorded for the SDW (Fig. 3F).

### Interconnection of root-shoot traits

**Figure 4:**
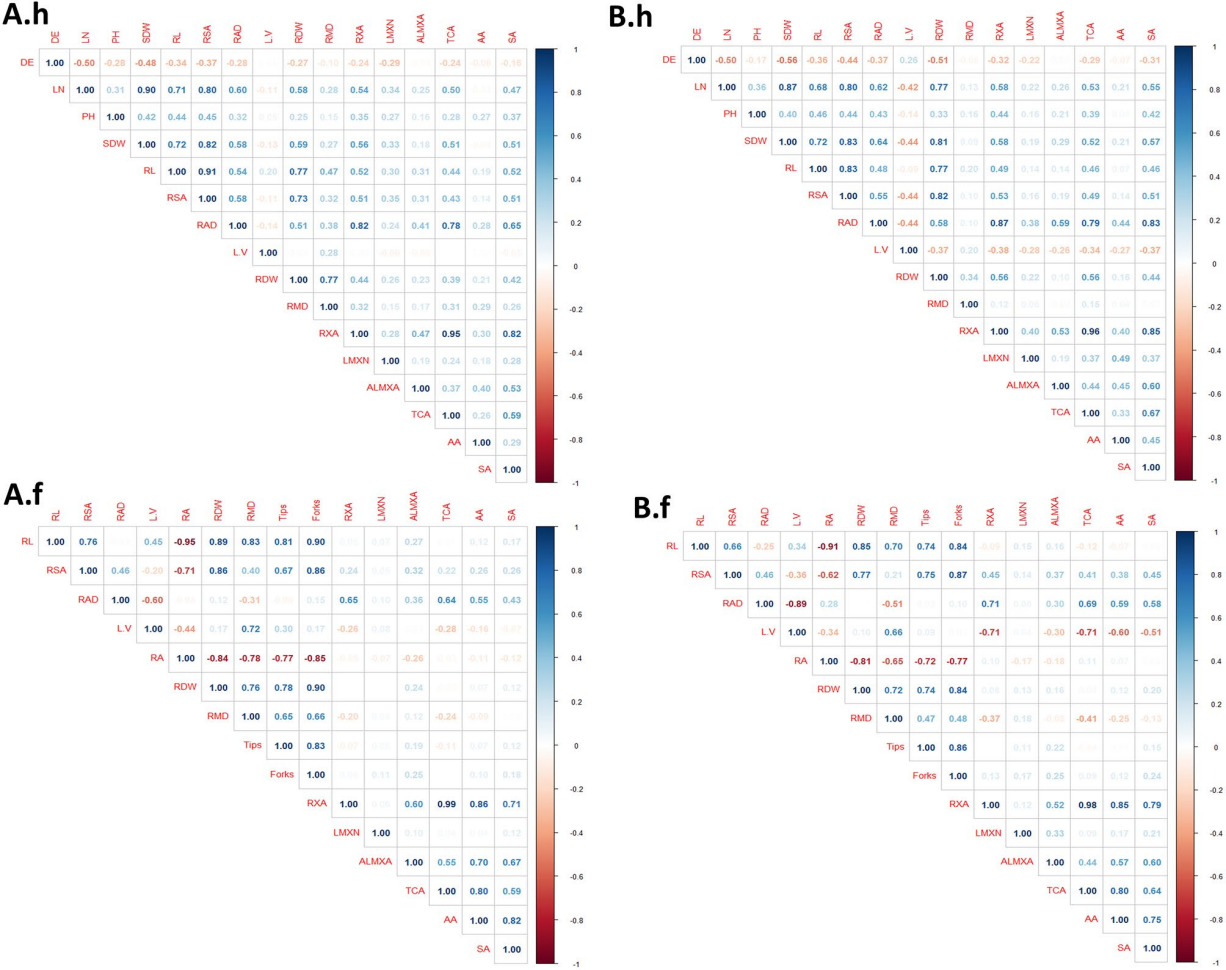
Correlation matrix for shoot morphological (only in hydroponic conditions; **A.h** and **B.h**), root architectural and anatomical traits in two groups of barley populations and their parental lines grown across two growing conditions. (**A**)—conventional and (**B**)—organic cropping systems. (**A.h**)—conventional under hydroponic, (**B.h**)—organic under hydroponic, (**A.f**)—conventional under field, and B.f—organic under field conditions. The color scale represents Spearman's ranked correlation coefficient. A larger circle size indicates a smaller *p*-value; blank cells represent that correlation was non-significant at *P* < 0.05. Most strongly associated traits are ordered and grouped in black boxes based on hierarchical clustering. See Table 1 for trait description.

We performed inter-trait correlation analysis to unravel association among root traits and in between root and shoot phenotypes (Fig. 4). Pearson correlation coefficient revealed significant correlations among root-shoot traits. LN, PH and SDW had strong positive associations with all root architectural traits under hydroponic conditions (*P* < 0.001, r =  > 0.30) in both CCS and OCS, while DE has negative association with all shoot traits (r = −0.17 to −0.48) (Fig. 4A.h,B.h). A consistent negative relationship was observed for L/V with shoot traits such as LN, PH and SDW and root morphological traits such as RL, RSA and RAD in both CCS and OSC populations (Fig. 4A.h,B.h). A strong negative association existed between RL and all shoot morphological, root architectural and anatomical traits in both populations, except for L/V where a weak negative (r = −0.09) association was displayed only in the OCS. Likewise, all above-ground traits and all root architectural traits exhibited significant positive associations with all root anatomical features in both groups with an exception for the AA (Fig. 4A.h,B.h). Moreover, correlation analysis revealed strong positive relationships in both groups of SDW and root dry weight (RDW) to all above-ground traits, below-ground traits including, RL, SA, and RAD, as well as in all root anatomical traits (Fig. 4A.h,B.h). This means that the growth of tissue and organ is proportional to the increase in total dry biomass. More importantly, we observed a significant positive correlation among most of the root morphological, architectural, and anatomical traits in both OCS and CCS adapted populations, with few exceptions such as L/V (Fig. 4A.h,B.h).

Similar to hydroponics, correlation analysis for both groups under field conditions showed highly significant positive and negative correlations (Fig. 4A.f,B.f). While a positive correlation was observed under hydroponics, RDW only showed to be a positive significant relationship with other root morphological traits and a weak positive relationship with anatomical traits in response to both CCS and OCS. Interestingly, correlation analysis revealed the important association of RA to other root architectural and anatomical features, and showed a strong negative association between RL (r =  > −0.90) and RDW (r =  > −0.80) (Fig. 4A.f,B.f) for CCS and OCS populations respectively, which means that narrower the angle of the nodal roots, the longer was the root system. The two root branching traits, the number of tips and number of roots forks which were known to be associated and dependent on the RL have a strong positive correlation reflected by r = 0.81 and 0.90 in CCS and r = 0.74 and 0.84 in OCS developed lines, respectively, while they have a significant negative correlation with RA (r = −0.72 to −0.77) in both barley groups. In addition, RA had also strong negative relationship to RDW contributing architectural traits including, RMD (r = −0.81 to −0.84) and L/V (r = −0.34 to −0.44). However, no positive associations were observed for RA and all root anatomical traits in both OCS and CCS populations (Fig. 4A.f,B.f).

### Allometry analysis

The correlation analysis identified interconnection among root and shoot-related traits. Therefore, we checked if these correlations can be explained by allometric relations (Tables 3 and 4).

**Table 3:** Summary of allometric analysis of root-shoot system traits under hydroponic condition.

Traits	DE	TN	LN	PH	SDW	RL	RSA	RAD	RV	L/V	RDW	SRL	RMD	R:S	RXA	ALMXA	TCA	AA	SA
DE	1	0.017	0.567	0.027	0.273	0.58	0.753	0	0.347	0.017	0.077	0.76	0.08	0.723	0.683	0.677	0.47	0.717	0.533
TN	0.017	1	0.15	1	0.88	1	0.523	0.997	0.893	1	1	1	1	1	1	1	1	1	1
LN	0.567	0.15	1	1	0.897	1	0.84	0.993	0.897	1	0.98	1	1	1	1	1	1	1	1
TPH	0.027	1	1	1	0	0.227	0.037	0.207	0.03	0.327	0.007	0.05	0.017	0.007	0.073	0.153	0.077	0.24	0.173
SDW	0.273	0.88	0.897	0	1	1	0.997	0.997	1	1	1	1	1	1	0.997	1	1	1	1
RL	0.58	1	1	0.227	1	1	1	1	1	1	0.943	1	0.997	1	1	1	1	1	1
RSA	0.753	0.523	0.84	0.037	0.997	1	1	1	0.95	1	0.977	1	1	1	1	1	1	1	1
RAD	0	0.997	0.993	0.207	0.997	1	1	1	0.853	0.517	0.923	0.83	0.687	0.863	0.73	0.623	0.507	0.47	0.933
RV	0.347	0.893	0.897	0.03	1	1	0.95	0.853	1	1	0.913	1	1	1	1	1	1	1	1
L/V	0.017	1	1	0.327	1	1	1	0.517	1	1	1	0.973	1	1	1	1	1	1	1
RDW	0.077	1	0.98	0.007	1	0.943	0.977	0.923	0.913	1	1	1	1	1	1	1	1	1	1
SRL	0.76	1	1	0.05	1	1	1	0.83	1	0.973	1	1	1	1	1	1	1	1	1
RMD	0.08	1	1	0.017	1	0.997	1	0.687	1	1	1	1	1	1	1	1	1	1	1
R:S	0.723	1	1	0.007	1	1	1	0.863	1	1	1	1	1	1	1	1	1	1	1
RXA	0.683	1	1	0.073	0.997	1	1	0.73	1	1	1	1	1	1	1	0.9	0.05	0.843	0.82
ALMXA	0.677	1	1	0.153	1	1	1	0.623	1	1	1	1	1	1	0.9	1	0.853	0.133	0.927
TCA	0.47	1	1	0.077	1	1	1	0.507	1	1	1	1	1	1	0.05	0.853	1	0.893	0.62
AA	0.717	1	1	0.24	1	1	1	0.47	1	1	1	1	1	1	0.843	0.133	0.893	1	0.69
SA	0.533	1	1	0.173	1	1	1	0.933	1	1	1	1	1	1	0.82	0.927	0.62	0.69	1

The *P*-values of allometric cross comparison of root morphological, anatomical and shoot traits. *P*-value < 0.05 indicate a significant allometric relation between the traits calculated by bootstrapping (boot = 300). See Table 1 for traits description.

**Table 4:** Summary of allometric analysis of root-shoot system traits under field condition.

Traits	RL	RSA	RAD	RV	L/V	RA	RDW	SRL	RMD	#Tips	#forks	RXA	ALMXA	TCA	AA	SA
RL	1	0.287	0.323	0.253	0.133	0	0.803	0.77	0.217	0.607	0.23	0.893	0.857	0.943	0.867	0.887
RSA	0.287	1	0.017	0.377	0.11	0.153	0.597	0.607	0.053	0.303	0.327	0.773	0.84	0.797	0.83	0.907
RAD	0.323	0.017	1	0.743	0.287	0.867	0.79	0.783	0.38	0.907	0.28	0.183	0.253	0.277	0.797	0.79
RV	0.253	0.377	0.743	1	0.05	1	0.987	0.81	0.913	1	0.993	0.977	0.98	0.957	1	0.987
L/V	0.133	0.11	0.287	0.05	1	0.963	0.99	0.997	0.847	0.923	0.97	0.863	0.993	0.85	0.91	0.987
RA	0	0.153	0.867	1	0.963	1	0	0.033	0.007	0.08	0.03	0.21	0.21	0.253	0.167	0.657
RDW	0.803	0.597	0.79	0.987	0.99	0	1	0.867	0.24	0.943	0.35	1	1	1	1	1
SRL	0.77	0.607	0.783	0.81	0.997	0.033	0.867	1	0.713	0.893	0.7	1	1	1	1	1
RMD	0.217	0.053	0.38	0.913	0.847	0.007	0.24	0.713	1	1	0.97	1	1	1	1	1
#Tips	0.607	0.303	0.907	1	0.923	0.08	0.943	0.893	1	1	0.823	0.947	0.94	0.963	0.967	0.667
#forks	0.23	0.327	0.28	0.993	0.97	0.03	0.35	0.7	0.97	0.823	1	1	1	1	1	1
RXA	0.893	0.773	0.183	0.977	0.863	0.21	1	1	1	0.947	1	1	0.463	0.12	0.977	0.047
ALMXA	0.857	0.84	0.253	0.98	0.993	0.21	1	1	1	0.94	1	0.463	1	0.927	0.983	0.387
TCA	0.943	0.797	0.277	0.957	0.85	0.253	1	1	1	0.963	1	0.12	0.927	1	0.803	0.033
AA	0.867	0.83	0.797	1	0.91	0.167	1	1	1	0.967	1	0.977	0.983	0.803	1	0.3
SA	0.887	0.907	0.79	0.987	0.987	0.657	1	1	1	0.667	1	0.047	0.387	0.033	0.3	1

The *P*-values of allometric cross comparison of root morphological, anatomical and shoot traits. *P*-values < 0.05 indicate a significant allometric relation between the traits calculated by bootstrapping (boot = 300). See Table 1 for traits description.

In the hydroponic environment, we observed a total of ten allometric relations, from which six were annotated to the PH. The PH was allometrically related to the SDW, the RSA, the RV, the RDW, the SRL, and the RMD (Table 3). Besides, the SDW was allometrically associated with the RSD. Furthermore, the TCA was related to the RXA. Finally, an allometry relationship was detected between the LMXN and AA (Table 3).

In the field experimental setup, we detected in total ten allometric relations (*P* < 0.05). The most significant allometry was observed for the RL and the RA (Table 4). Besides, the RA was allometrically related to the RDW, the SRL, the RMD, and the number of root forks. Furthermore, the RAD growth was significantly related to the growth of RSA. Finally, we also observed an allometric relationship between TCA and the SA (Table 4).

**Figure 5:**
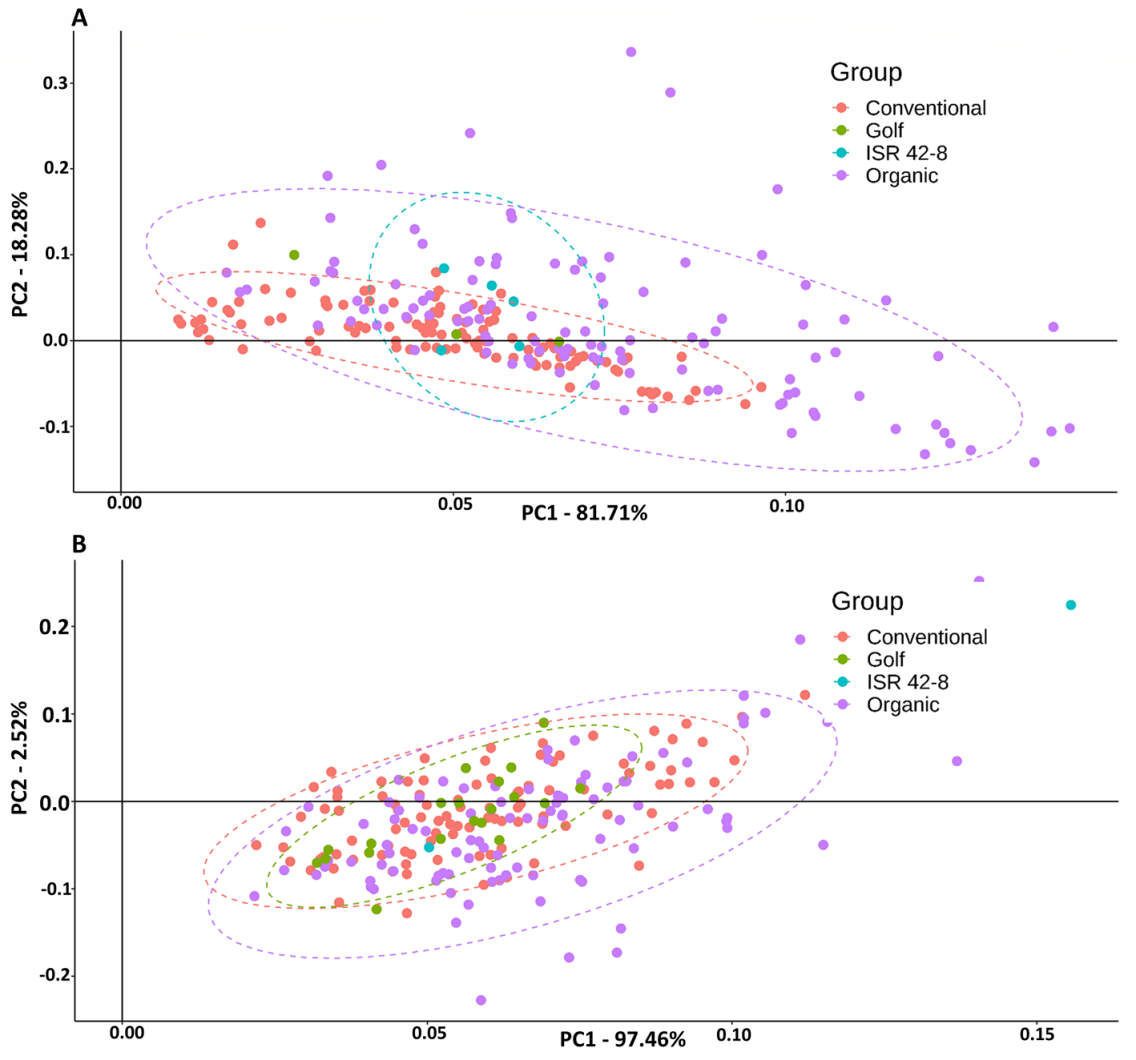
The principal component output of the root morphology assessment in a hydroponic (**A**) and a field-based environment (**B**). The first two components are plotted, with the first PC on the *x*-axis and the second PC on the *y*-axis. The colors differentiate the conventional (magenta) and organic (purple) systems, and the wild donor (ISR 42-8, turquoise) from the cultivar (Golf, green). Each point represents an individual genotype. In (*A*), 150 genotypes were tested from the organic and conventional systems, while 100 genotypes each were tested in (*B*). Dashed ellipses are plotted based on the points if the sample size was sufficient. Both experiments illustrate higher genetic variance in the organic system as compared to the conventional system.

Root system divergence exists in barley populations. Based on the root phenotypic traits, PCA was conducted to highlight variations between parental lines as well as OCS and CCS populations (Fig. 5). Lines that evolved in the OCS environment tend to have a higher variance compared to CCS adapted lines in both experimental setups (Hydroponics and field, Fig. 5A,B). The majority of variance is explained by the first component (PC1) (81.71% hydroponics, 97.46% field), while the second component (PC2) explains 18.28% and 2.52% for hydroponics and field experiments, respectively (Fig. 5). The principal components (PCs) of both environments highlight a similar range of values and extension of points.

### Comparison of root phenotypes with the genomic background

**Figure 6:**
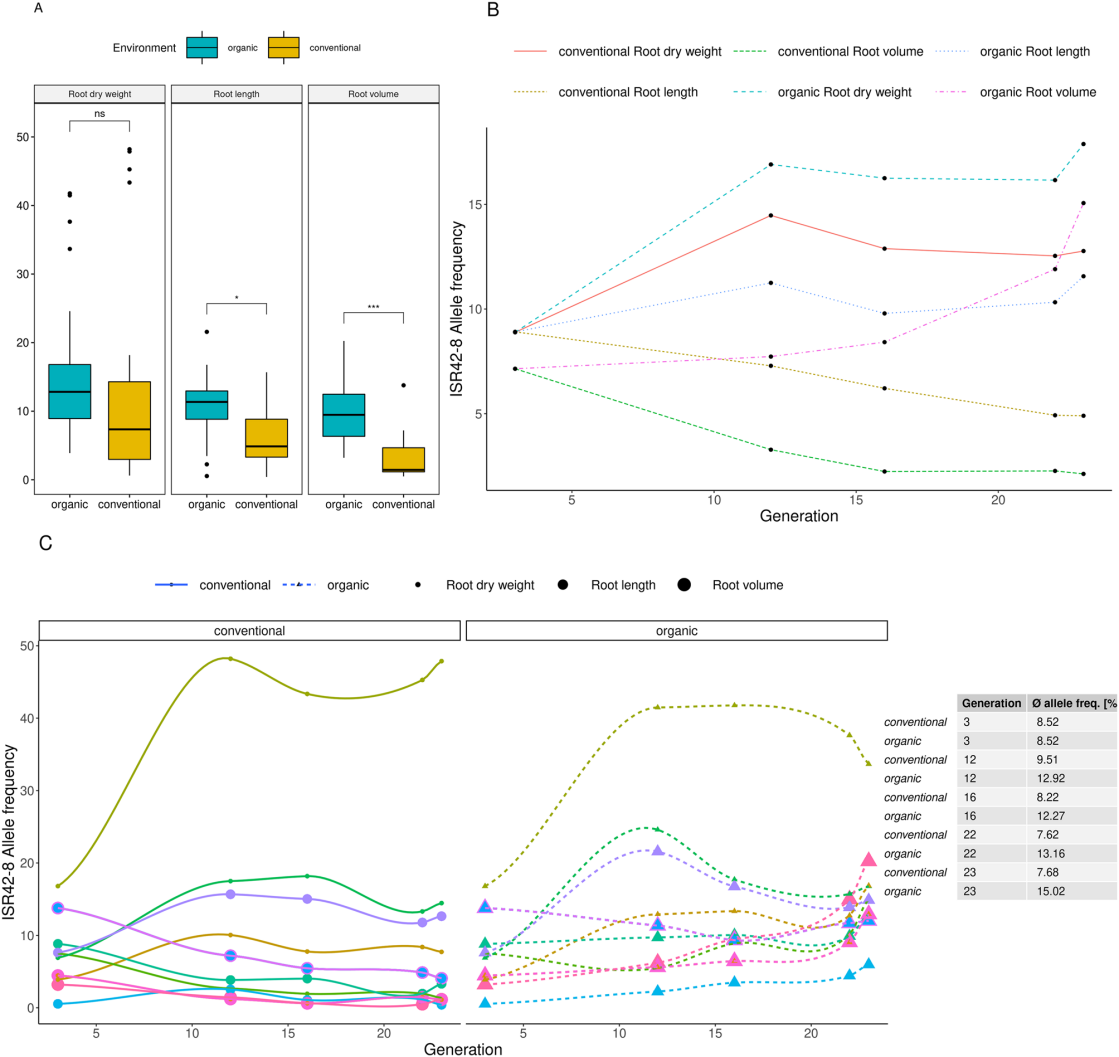
The allele frequency pattern dissection of 18 selected QTL loci related to root architecture traits for both organic and conventional populations in 5 generations (F3, F12, F16, F22, F23). Genomic data derived from Schneider et al. (2021). (**A**) Boxplot of the ISR42-8 allele frequencies for the trait categories root dry weight, root length, and root volume. Stars indicate the significance level of adjusted p values (Bonferroni adjustment) between the environments. The data of all generations are included in this significance test. (**B**) Line plot showing the development of the ISR42-8 allele frequency for the same three categories, separated by the environments across the generations. (**C**) similar to B, but showing the evolution of the ISR42-8 allele frequency across the generations for each QTL locus, separately. Table—indicates the median allele frequency across all 18 QTL loci generation and environmentally wise.

To support the validity of the observed phenotypic variations between the OCS and CCS groups, we inspected the allele frequency patterns of root developmental-related QTL, as described by^35^. For that, we utilized the genetic and adaptation data of the two populations in the earlier generations, as described by^26^. Eighteen loci, clustered in three phenotype classes: root dry weight, root length, and root volume were selected and compared between the organic and conventional populations in the starting (BC_2_F_3_) and four later generations (12, 16, 22 and 23) for their ISR42-8 allele frequency levels. We observed significantly higher ISR42-8 allele frequencies for root length and root volume in the organic population (p_root length_ = 0.015; p_root volume_ < 0.001, Bonferroni corrected T-test, Fig. 6A) that showed evidence of increase, even when considering all generations (F_3_ = 0.09; O_23_ = 0.12). The opposite is true for the conventional system (F_3_ = 0.09; C_23_ = 0.06). Contrasting, both organic and conventional populations show a higher ISR42-8 allele frequency for the root dry weight-related alleles (Fig. 6B, F3 = 0.09, O_23_ = 0.18, C_23_ = 0.13). When investigating all 18 loci separately, it becomes apparent that for QTL locus Qrdw.S42IL.1H, affecting the root dry weight, the ISR42-8 allele frequency increased up to 50% in both environments (Fig. 6C).

## Discussion

Here we report that after 20 years of judicious natural adaption towards OCS and CCS, barley populations segregated in root phenotypes. Variation of root system architecture is regarded as a significant aspect of plants' adaptability in response to fluctuating growing conditions^5,12,36,37^. These differences are critical parameters that could influence plants' competitive ability in searching for unevenly distributed soil resources and hence a predictor of plants' adaptation to various growing conditions^9,10,38^.

Trait evaluations were performed in two different test environments (hydroponic and field) with the intention to produce redundant and additional phenotypic information. This resulted in a better resolution for some root traits under both hydroponics and field experiments. We performed the experiment without additional replications of each line, as the overall objective was to determine phenotypic variation between the CCS and OCS populations and not between the genotypes within the populations. Both organic and conventional genotypes grown under hydroponics and field conditions were harvested identically, which results in an identical bias among the groups. Nevertheless, the testing under field conditions could result in an increased underestimation of root architectural parameters. Therefore we complemented the field trial with a hydroponic experiment, where the entire root system morphology could be estimated. As already shown in root morphological trials in *Brassica*, the root development in the seedling stage is a reliable indicator for the following development stages^39^. We hypothesized that this is also true in barley.

Another important reason that should be mentioned is the differences among root parameters acquired in both growing conditions. Equal and consistent access to water, nutrients, oxygen and other physical factors such as temperature and light were provided continuously under hydroponics growing conditions. Contrastingly, plants were exposed to fluctuating environmental factors, and competition of soil resources occurred when grown under field conditions. These factors were reported to influence root growth ability directly or indirectly^40,41^. Another factor influencing the root architecture is the spacing of the plants in the field experiment and the horizontal growth limitation in the hydroponics experiment. Both result in limitations when trying to assess some phenotypes, like the absolute root length or the root angle.

The two parental genotypes used to establish the populations are highly variant in terms of their root architecture^35^. While ISR42-8 is a wild form, adapted to its local arid low input environment, Golf is a former elite cultivar, selected to produce high yields in humid climates under nutrient supplementation. These two divergent genetic backgrounds were partly reflected in the phenotypes of the subset of the lines selected from OCS and CCS populations (Figs. 1 and 2). While CCS were supplemented with mineral nitrogen fertilizers and therefore not exposed to an environment of nutrient deficiency, the OCS population was not supplemented with mineral fertilizer (Table S1B), but instead with manure to two of the seven crops in the crop rotation. The mixed soil nutrient profile of all seven organic crops indicates that the fertilization with manure resulted in increased nutrient levels of phosphorus and potassium. Although lower, also the conventional system indicated more than sufficient levels of phosphorus, potassium, and magnesium. Still, the position of spring barley in the crop rotation, being the 4th crop grown after the application of manure following potato (manure applied), field bean, and winter rye, will either result in (I) a soil nitrogen depletion or (II) a nitrogen leaching in deeper soil layers. For the second scenario, the application time point of manure is crucial. In this long-term experiment, manure was applied in the fall. According to^42^, the main mineralization activity will happen the following year in late spring and summer. This might lead to the fact that potatoes cannot utilize a major part of the mineralized nutrients, leaving minerals unused in the soil matrix. The following crop, field bean, is sown in early spring the following year, leaving the field with no catch crop over the winter. This might promote nitrogen leaching to deeper soil layers. Additionally, the following crop in the rotation is the field bean, a legume, which does not depend on external nitrogen due to its ability to build symbiosis with rhizobium bacteria. Concluding, these two years might promote the leaching of nitrogen towards deep soil layers. These aspects make it apparent that plants grown in the organic system might need to build a more diverse root system to reach all required soil nutrients.

Similarly to this hypothesis, we observed that the variations in RL, L/V, and RA in the OCS population were more pronounced compared to the CCS (Fig. 1A–C). Similar to the RL, the increase of RL in the OCS line is likely associated with the genetic background of ISR42-8, so it could be assumed that the root grows towards deep soil layers to capture deep soil resources^6,43,44^. Deep and steeper roots lead to improved access to soil resources and consequently improve yield in some crops such as rice^45^, wheat^46^, and nitrogen uptake in maize^47^. Moreover, longer roots with narrow RA, higher RMD and L/V, and bigger RSA were recorded among lines that originated from OCS (Fig. 1; Table 2). This data, especially from the hydroponic experiment, indicated that these lines potentially target deep soil layers. The results presented are substantiated by the recent study^48^. They evaluated the early seedling traits of evolving winter wheat composite cross populations (CCPs) and reported that over time CCPs exposed to the organic management system for several generations (F_11.1_) showed considerably higher root performance as displayed by its longer seminal RL, narrow system and higher RDW.

In addition, we performed a root trait-focused analysis of the genomic data collected for previous generations of these two populations, presented by^26^. From this data, it can be concluded that (I) the ISR42-8 allele frequency has changed over the generations in both systems and has resulted in (II) a significantly higher ISR42-8 allele frequency in the organic population for root length and root volume-related QTL regions (Fig. 6). It appears that some wild form alleles at root related QTLs positively affect the fitness of the individual lines in both populations—so that these alleles are positively selected across generations. Concluding, we see significantly higher ISR42-8 allele frequencies in the organic population when considering selected root length and root volume-related QTL regions, supporting the observations made in the phenotyping of the 24th generation.

Furthermore, important features of root anatomical adjustments between lines originated in two different management systems were significantly variant, mostly recorded in LMXN, TCA, AA, and RXA (Fig. 2). RXA is mainly associated with root thickness, which is relatively expressed in the subsequent thickness of the root cortical and pith tissue layers^49^. It is also well reported that the thick root diameter is associated with large cortex and aerenchyma size^50,51^. In this investigation, barley lines that produce a bigger RAD also have bigger RXA, TCA, and AA (Table 2, Fig. 2). Likewise, conventionally selected lines that were found to have bigger RAD and RV compared to those under the organic system indeed also produce comparatively bigger size for root anatomical traits such as RXA, ALMXA, TCA, AA, and SA than barely lines evolved under the organic system (Table 2). Comparing the two barley populations, organically managed lines had superior LMXN than conventionally managed lines (Fig. 2A,h,A,f).

Our results indicated that barley lines with thinner roots due to decreased TCA or SA and had a smaller ALMXA and tend to have a LMXN, which is particularly displayed by lines evolved under the OCS. This might indicate a variation in the development of these root anatomical organs between the cropping systems. The effect could either result from a long-term farming practice-depended variation or from the exposed environment itself. Under hydroponic conditions, water supply was never in shortage, while it is a more limiting factor under field conditions. Therefore, the observed patterns could be an indication of increased metaxylem size, which ultimately is a reflector of water stress response^52^. In contrast, the higher LMXN in the OCS lines might be an indication of fewer lines suffer from water limitations. A relevant correlation between the RL and the LMXN could not be observed, most likely due to the impossibility of harvesting the root as a whole under field conditions. However, several reports noted that water-conducting tissue tends to be smaller, and plants tend to develop more LMXN to compensate for smaller diameters^51,53,54^. Narrow xylem diameter, particularly in seminal roots, was reported to optimize soil water from deep soil profile for plant utility, especially during maturation^53^, whereas broad xylem diameter in deep-growing roots was known to also improve the exploration of available water within deep soil horizons^55^. The cortex, which is composed of parenchyma tissue, plays a crucial role in regulating water and nutrient transport via the apoplast and symplast pathways^56^. A lower TCA was recorded by OCS (Fig. 2C,f) that might be associated with a higher nutrient and energy use efficiency. As OCS lines already tend to produce longer RL, a thick cortical area would extend their fitness disadvantages in terms of energy allocations away from the seeds^57^. Moreover, aerenchyma (air cavities that consist of gas-filled spaces) plays a vital role in the long-distance transport of oxygen from the shoots to the root tips which is particularly important in the adaptability and survival of plants, particularly under stressful growing condition^54^. Root aerenchyma has been related to improving crop growth in maize plants grown under water-deficit conditions^55,58^.

Additionally, allometric associations were observed between root traits and plant size. The most relevant is the RA to RL, RDW, and number of root forks (Tables 3 and 4). These traits, especially RA might be a good candidate for root breeding, as it determines the entire root system development. Nevertheless, the genetic background of all lines is limited to two inbred lines, making extrapolations of such allometric observations difficult. Besides the RA, only the PH indicated six allometric relations. This might be associated with the rapid growth in the early growth stage and therefore, the allometry of the PH might be overestimated. Moreover, an increased phenotypic variation was observed in the OCS lines across various root morphological and anatomical traits (Fig. 5 and Table S4). This heterogeneity might originate from genotypes inhabiting different ecological niches within the population, e.g. some genotypes might have more shallow roots to capture the upper soil layers, while others have predominantly developed higher RL to reach deep soil layers^44^.

In conclusion, the tested lines from the organic adapted population were observed to have an adaptive root system, represented by longer roots with a narrow root angle, larger root surface area, root density, and a thinner root size with a higher late metaxylem number. In comparison to the organic population, lines adapted to conventionally treated lines tend to have a shorter and wider root architecture, which maintain a thicker root size but has fewer late metaxylem number (Figs. 1 and 2). Moreover, long-term selection by organic farming system contributed to substantial genetic variation in root system attributes observed under both hydroponic and field conditions (Fig. 5). The allele frequency patterns, derived from genomic data of previous generations, supported the observations made by the root phenotyping (Fig. 6). The deep rooting patterns of organic lines can be beneficial in terms of soil erosion reduction^59^, drought tolerance^60^, lodging tolerance^61^, and increased nutrient use efficiency and preserved leaching^62^. Furthermore, a larger variation of root morphological characteristics was observed in the organic population. Overall, our results indicate the different requirements of root growth between OCS and CCS, which may provide additional insight into the nutrient use efficiency. Varieties with the described root phenotypes could serve as valuable genetic resources in future breeding.

## Supplementary Information

Supplementary Information.

## Data Availability

The authors confirm that the data supporting the findings of this study are available from the corresponding author, upon request.
