# Supplementary material for: Natural selection under conventional and organic cropping systems affect root architecture in spring barley
Source: Sci Rep. 2022 Nov 22;12:20095. doi: 10.1038/s41598-022-23298-3 (PMC9684413; doi:10.1038/s41598-022-23298-3)
Supplement: Supplementary file 1 — Supplementary Information. [file 41598_2022_23298_MOESM1_ESM.pdf]

Supplementary information

Title: Natural selection under conventional and organic cropping systems affect root architecture in spring barley

Author list:
Md. Nurealam Siddiqui, Michael Schneider, Marissa Barbosa, Jens Léon and Agim Ballvora

This PDF file includes:

Supplementary Figure S1: Workflow overview of the experiment.

Supplementary Figure S2: Shoot morphological traits measured in two groups of barley evolved from long-term selection under conventional and organic farming system and their parent lines Golf (cultivated variety) and ISR 42-8 (wild-type).

Supplementary Table S1: Field experiment trail layout (A) and treatment (B) of organically and conventionally farmed environments.

Supplementary Table S2: Average soil p^H and nutrient profiles of organic and conventional cropping systems in the period from 2014 to 2020 and average precipitation data of field experiment in 2020.

Supplementary Table S3: Comparison of parental root phenotypes under field and hydroponic evaluation.

Supplementary Table S4. Test for homogeneity of variance between two barley populations evolved under conventional and organic cropping systems across two different growing conditions.

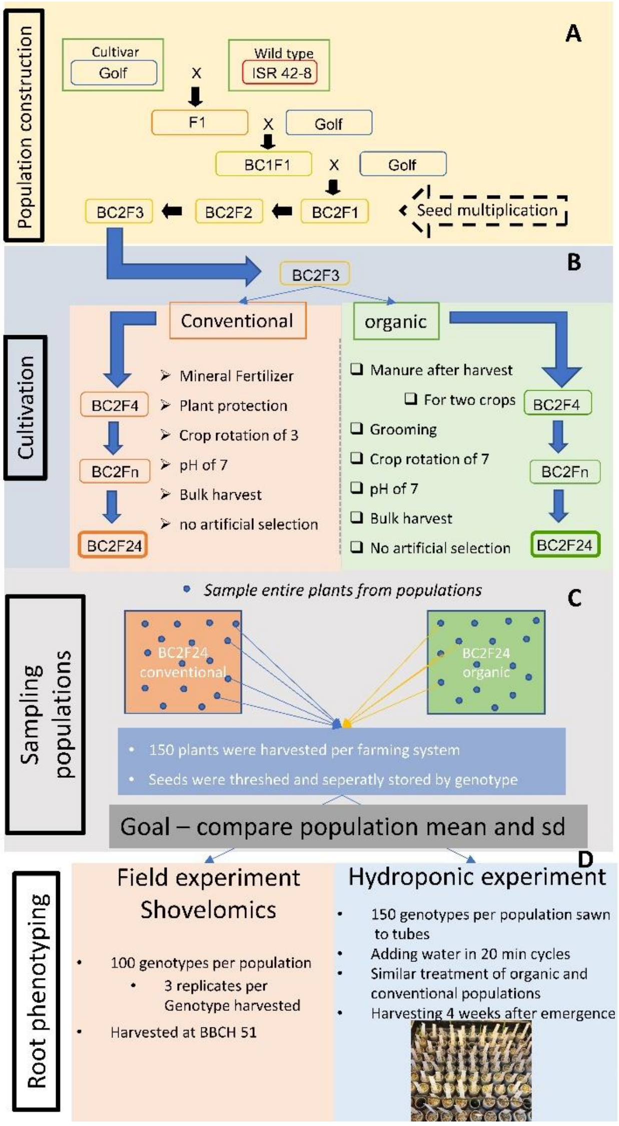

Fig. S1: Workflow overview of the experiment. (A) Crossing and establishment of the spring barley populations. (B) The field experiment layout with generations and treatments. Seeds from previous years were used to establish the next generation. (C) Selection of 150 genotypes randomly, harvested as an entire plant from the organic and conventional evolved BC2F24 generation and (D) Root phenotyping of both populations in two different environments (hydroponics and field experiment).

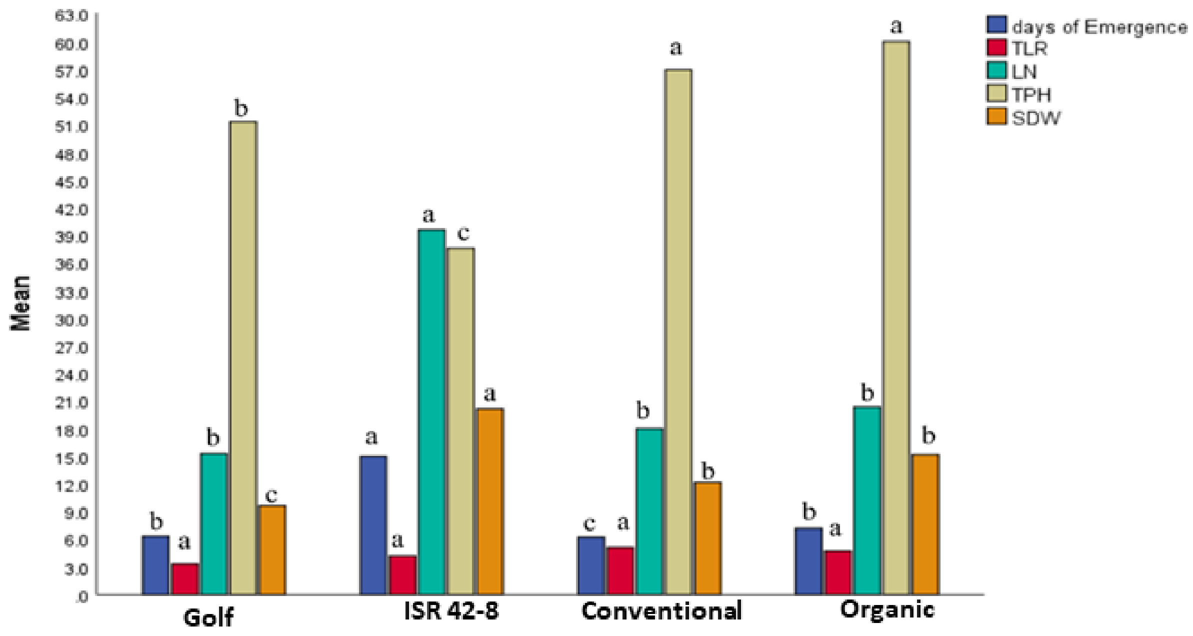

Fig. S2: Shoot morphological traits measured in two groups of barley evolved from long term selection under conventional and organic farming system and their parent lines Golf (cultivated variety) and ISR 42-8 (wild-type). Groups with the same letters are not significantly different (Tukey HSD test ($P \leq 0.05$). TLR, tiller number; LN, leaf number; TPH, plant height (cm); SDW, shoot dry weight (gm).

Supplementary Table S1: Field experiment trail layout (A) and treatment (B) of organically and conventionally farmed environments. A – Crop rotation of organic and conventional farming. The spring barley populations of interest are illustrated in blue and rotate to the right in their blocks in the next year. The conventional farming system consists of a three-crop rotation, the organic farming system of seven crops. The barley populations always remained in their row, while the conventional population rotates in the first to third column, and the organic population in the fourth to tenth column. **B** – treatments in both environments; [WW – winter wheat, SB – spring barley, WRa – winter rape seed, Pot – Potato, AB – common bean, WR – winter rye, OAT – spring oat].

A

**conventional**				**organic**						
3 m border				3 m border						
**WRa**	**WW**	**SB**		**AB**	**WRa**	**SB**	**Oat**	**WR**	**WW**	**Pot**
9 m border				9 m border						
**SB**	**WRa**	**WW**		**WR**	**Pot**	**Oat**	**AB**	**WW**	**SB**	**WRa**
9 m border				9 m border						
WW	***SB P1***	***WRa***		**SB**	**Oat**	**WRa**	**WR**	**Pot**	**AB**	**WW**
9 m border				9 m border						
**WRa**	**WW**	**SB**		**Pot**	**AB**	**WR**	***SB P1***	**WRa**	**WW**	**Oat**
3 m border				3 m border						
										9 m

			Environment	
**B**			**Organic**	**Conventional**
Fertilizer		Type	Manure	Minerals
		Times of Application	once per year for Potato and Rape seed	Booting and Flowering Stage
		Amount added	200 dt/ha – 156kg N, 78 kg P2O5, 374 kg K2O, 36 KG MgO,*	80 – 100 kg N by calcium ammonium nitrate*
Growth Control		Seed Treatment	Seeds coated for sowing	Seeds coated for sowing
		Growth Regulation	none	on demand²
		Fungicide Treatment	none	on demand²
		Competition Control	groom between rows	Herbicide on demand²
System Managed		Crop Rotation	Rape Seed – Winter Rye – Winter Wheat – Acre Beans – Oat – Spring Barley – Potato	Rape Seed – Winter Wheat – Srping Barley
		Soil Balance	pH, Nmin, Pmin, organic matter	pH, Nmin, Pmin, organic matter
		pH Adjustment	lime to pH of 7	lime to pH of 7
		* – Phosphate fertilization was not required due to high saturation level of the soil		
		2 - only products with a valid registration certificate of the year of use were utilized		

Supplementary Table S2: Average soil p^H and nutrient profiles of organic and conventional cropping systems in the period from 2014 to 2020 and average precipitation data of field experiment in 2020. Mixed spoil sample taken from all soil layers down to 90 cm below ground. Sampling was performed in autumn before sowing of winter wheat (part of the crop rotation). Standard deviation additionally provided for the adaptation experiment. Second part of the table indicates the precipitation data of the field experiment 2020.

**Cropping systems & Field experiment**	**Soil p^H**	**Soil nutrient profiles**			
		**P_2O_5 (mg)**	**K_2O (mg)**	**MgO (mg)**	**Organic matter (%)**
Conventional	6.86±0.1	30.0±3.54	22.0±0.7	8.0±0	1.74±0.15
Organic	6.92±0.1	35.3±3.2*	28.0±2.45*	8.8±0.45	1.72±0.16*
Field experiment 2020	6.5	16.0	16.0	8.0	2.7
**Precipitation (mm)**					
Experiment	Min	Max	Average	Sum	
Field experiment 2020	0.00	39.2	1.34	491.79	

*= increased across the generations

Supplementary Table S3: Comparison of parental root phenotypes under field and hydroponic evaluation**.**

	**Field**								**Hydroponics**							
	**Confidence interval**				**Average**		**Standard deviation**		**Confidence interval**				**Average**		**Standard deviation**	
**Traits**	*Difference**	*lower*	*upper*	*adj p value*	*ISR 42-8*	*Golf*	*ISR 42-8*	*Golf*	*Difference**	*lower*	*upper*	*adj p value*	*ISR 42-8*	*Golf*	*ISR 42-8*	*Golf*
RL	505.664	357.665	653.663	0.000	754.665	249.001	60.507	46.314	174.356	-1233.25	1581.970	0.989	1308.866	1134.510	185.745	638.493
L:V	2.913	-120.670	126.497	1.000	158.710	155.797	46.457	41.310	74.252	-579.164	727.669	0.991	825.153	750.900	267.253	158.324
RMD	0.076	-0.215	0.367	0.906	0.310	0.234	0.028	0.092	-0.013	-0.188	0.162	0.998	0.196	0.209	0.046	0.059
RA	-11.500	-28.044	5.044	0.276	81.000	92.500	2.828	3.980	-	-	-	-	-	-	-	-
#Forks	2661.551	1123.187	4199.914	0.000	4343.835	1682.285	3093.118	600.955	-	-	-	-	-	-	-	-
#Tips	862.039	97.777	1626.301	0.020	1817.165	955.126	1312.624	227.148	-	-	-	-	-	-	-	-
RSA	83.573	41.834	125.311	0.000	153.595	70.023	70.689	16.367	110.766	-77.921	299.454	0.428	269.977	159.210	95.773	94.097
RAD	0.252	-0.124	0.628	0.309	1.185	0.933	0.106	0.153	0.002	-0.202	0.206	1.000	0.403	0.401	0.040	0.025
SRL	-174.057	-700.004	351.890	0.827	569.050	743.107	18.427	222.645	486.870	-11179.6	12153.3	1.000	4121.683	3634.813	497.206	253.317
RV	3.234	1.922	4.545	0.000	4.910	1.677	1.061	0.572	0.149	-2.308	2.606	0.999	1.785	1.636	0.773	1.012
RDW	1.131	0.579	1.682	0.000	1.505	0.375	0.191	0.163	0.015	-0.640	0.671	1.000	0.322	0.307	0.063	0.166
R:S	-	-	-	-	-	-	-	-	-0.076	-0.460	0.308	0.957	0.260	0.335	0.051	0.043
RXA	0.273	-0.164	0.709	0.371	1.390	1.118	0.085	0.174	0.041	-0.176	0.259	0.961	0.368	0.326	0.033	0.087
TCA	0.157	-0.220	0.533	0.704	1.100	0.944	0.071	0.177	-0.041	-0.197	0.114	0.902	0.193	0.234	0.037	0.058
AA	0.024	-0.176	0.224	0.990	0.396	0.372	0.007	0.106	-0.001	-0.007	0.006	0.996	0.033	0.034	0.002	0.002
ALMXA	0.000	-0.001	0.001	0.855	0.002	0.001	0.000	0.000	0.000	-0.003	0.002	0.975	0.003	0.003	0.001	0.001
SA	0.118	0.025	0.210	0.006	0.360	0.243	0.042	0.025	0.021	-0.061	0.103	0.912	0.113	0.092	0.024	0.032
LMN	5.200	3.699	6.701	0.000	10.000	4.800	0.000	0.616	1.467	-0.262	3.195	0.127	5.800	4.333	0.837	0.577
DE	-	-	-	-	-	-	-	-	8.667	6.267	11.066	0.000	15.000	6.333	0.000	1.155
PH	-	-	-	-	-	-	-	-	-13.733	-27.691	0.225	0.056	37.600	51.333	4.775	11.240
LN	-	-	-	-	-	-	-	-	24.267	6.211	42.322	0.003	39.600	15.333	4.827	7.638
TN	-	-	-	-	-	-	-	-	0.867	-4.029	5.762	0.968	4.200	3.333	0.837	1.155
SDW	-	-	-	-	-	-	-	-	0.291	-1.344	1.926	0.967	1.258	0.967	0.235	0.606

Tukey HSD pairwise comparison for root morphology, anatomy and shoot-related traits at 95% confidence level showing the difference between group means (second column) and the adjusted *p*-value (fifth column) for all root traits (first column) between the ISR 42-8 and Golf. Besides, the average value and standard deviation of the values for ISR42-8 and Golf are illustrated. The stable is split in two main windows – one for field, the other for hydroponic experiment. Coloring indicates significant traits (gray) and higher average (blue) and standard deviation values (green). *ISR 42-8 always as first value - negative values are related to smaller in ISR 42-8. See Table 1 for trait description.

Supplementary Table S4: Test for homogeneity of variance between two barley populations evolved under conventional and organic cropping systems across two different growing conditions.

Traits	Hydroponics					Field				
	Organic		Conventional		*P*-value	Organic		Conventional		*P*-value
	Mean	SD	Mean	SD		Mean	SD	Mean	SD	
RL	1803.1	279.2878	1218.973	188.611	0.0001	300.233	42.46293	277.949	74.65156	0.624
RSA	215.322	121.6886	186.857	83.37233	0.0041	84.251	14.67385	79.231	18.9096	0.138
RAD	0.399	0.118322	0.407	0.1	0.0717	0.875	0.631664	0.964	0.197484	0.733
RV	2.337	1.530033	2.121	1.052616	0.0009	1.691	1.528725	1.923	0.681175	0.68
L/V	928.936	455.4955	611.843	236.8789	0	199.925	30.47845	159.505	51.3938	0.003
RDW	0.463	0.337639	0.452	0.360555	0.484	0.536	0.680441	0.505	0.258844	0.058
SRL	5652.045	165.5584	4180.793	108.1835	0.038	706.182	75.18008	642.498	74.23635	0.0039
RMD	0.196	0.07746	0.199	0.104881	0.002	0.332	0.442719	0.27	0.118322	0.0614
RA	-	-	-	-		87.143	10.28	90.949	6.915345	0.0003
RXA	0.313	0.122474	0.308	0.109545	0.466	0.92	0.559464	1.053	0.236643	0.8134
LMXN	5.282	0.943398	5.54	0.935949	0.569	5.133	2.29826	4.755	0.732803	0.5182
ALMXA	0.003	0	0.003	0	0.383	0.002	0.054772	0.002	0	0.005
TCA	0.207	0.009	0.203	0.006	0.118	0.767	0.038	0.891	0.044	0.4279
AA	0.036	0	0.041	0	0.04	0.287	0.01	0.341	0.012	0.4133
SA	0.106	0.0025	0.104	0.0021	0.369	0.152	0.004	0.161	0.002	0.0394

See Table 1 for trait description. SD, standard deviation.
